# Loss of function mutation in a mitochondrial chaperone protein leading to dyregulated ROS production, and autoinflammatory disease in a single kindred

**DOI:** 10.1186/1546-0096-13-S1-O1

**Published:** 2015-09-28

**Authors:** A Standing, D Eleftheriou, C Paisan-Ruiz, D Rowcenzio, Y Hong, E Omoyinmi, P Woo, P Hawkins, H Lachmann, N Klein, P Brogan

**Affiliations:** 1 UCL Institute of Child Health, IIIP, London, UK; 2 UCL Institute of Neurology, London, UK; 3 UCL Royal Free Hospital, National Amyloidosis Centre, London, UK; 4 University College London, London, UK

## Introduction

Reactive oxygen species (ROS) are known to have many roles in the propagation of the inflammatory process. Indeed increased leucocyte ROS have been implicated in a number of autoinflammatory diseases (TRAPS, FMF, CAPS and FCAS2).

## Objective

To use genetic mapping, next generation sequencing and functional studies to identify the genetic cause for a severe unclassified autoinflammatory disease mimicking Wegener's granulomatosis in a consanguineous family.

## Patients and methods

Three affected children in a Pakistani family suffered from a severe and unusual autoinflammatory syndrome, presenting in the first year of life with recurrent fevers, erythema nodosum-like rash, severe oromucocutaneous ulceration, systemic inflammation, and massively elevated serum IgD, without mutation in MVK. One of the affected children also suffered from multifocal sterile osteomyelitis with bony lytic lesions and died at age 12 months from bronchopneumonia, and acute cervical myelopathy from cervical vertebral collapse. The two older children were resistant to treatment with corticosteroids, colchicine, several different DMARDs, anakinra and infliximab. Both were cured by allogeneic haematopoietic stem cell transplantation (HSCT) in their teenage years and remain well and off all treatment approximately 6 years later. We performed homozygosity mapping in the three affected siblings, two unaffected siblings and their unaffected parents; followed by targeted capture and re-sequencing of an identified region of homozygosity. To study protein function, we used small interfering and short hairpin RNA knockdowns in THP1 cells. THP1 cell lines were also generated over-expressing wild-type and mutant protein harbouring the same missense variation identified in the patients. ROS levels were measured by flow cytometry and production by electron spin resonance (ESR). Co-localisation studies were conducted in HEK-293T cells and peripheral blood mononuclear cells using confocal microscopy.

## Results

Within the 5Mb region identified from the homozygosity mapping; a missense variant of interest in a mitochondrial chaperone-like protein was discovered. This segregated with disease in the family. This variant was rare or absent in ethnically matched and other healthy controls. Knockdown of this gene in macrophage-like THP1 cells led to increased mitochondrial ROS production; whilst wild-type protein overexpression led to reduced levels of mitochondrial ROS, which was not observed with mutant protein overexpression.

## Conclusion

We describe a novel monogenic autoinflammatory disease caused by a loss-of-function mutation in a mitochondrial chaperone protein, leading to dysregulated mitochondrial ROS production and severe autoinflammatory phenotype, and cured with allogeneic HSCT.

